# Combined effects of transition metal nitrogen and graphene nanoribbon edge active sites on the oxygen reduction reaction catalytic performance of metal–N–carbon-based catalysts†

**DOI:** 10.1039/d4ra07513g

**Published:** 2025-06-20

**Authors:** Xiao Hong Chen, Qiao Wu, Yupan Zhang, Junchao Xiong, Di Ma, Xiaoyu Xie, Jun Lin, Ying Wu, Hongjie Meng

**Affiliations:** a Institute of Energy Power Innovation, North China Electric Power University Beijing 102206 China yingwu2000@hotmail.com; b School of Energy, Power and Mechanical Engineering, North China Electric Power University Beijing 102206 China; c School of New Energy, North China Electric Power University Beijing 102206 China; d School of Energy Science and Technology, Longzihu New Energy Laboratory, Henan University Zhengzhou 450046 PR China menghongjie@sjtu.edu.cn

## Abstract

Metal–nitrogen–carbon (metal–N–C)-based catalysts with optimized local and external structures have received considerable attention owing to their improved activity and stability for the oxygen reduction reaction (ORR) in fuel cells. Abundant well-defined active sites on catalysts effectively enhance ORR performances. Herein, the Fe/Co–nitrogen–carbon-graphene nanoribbons (Fe/Co–N–C-GNRs) hybrids were obtained through the *in situ* growth of Fe/zeolitic imidazolate framework-67 particles on the surface of graphene oxide nanoribbons. The Fe/Co–N–C-GNRs exhibit a high electrocatalytic activity for the ORR (onset and half-wave potentials of 0.95 V and 0.83 V, respectively) and high durability, which are superior to those of 20 wt% Pt/C, suggesting Fe/Co–N–C-GNRs provide Fe–N_*x*_, Co–N_*x*_, and FeCo–N_*x*_ and GNR edge active sites. The Fe/Co–N–C-GNRs are excellent functional electrocatalytic catalysts exhibiting significant potential for fuel cell, chlor-alkali industry and lithium–oxygen battery applications.

## Abstract 2

Fe/Co–N–C-GNRs exhibit a high electrocatalytic activity for the ORR (onset and half-wave potentials of 0.95 and 0.83 V, respectively) and high durability, due to the combined effects of Fe–N_*x*_, Co–N_*x*_, FeCo–N_*x*_, and GNR edge sites.

## Graphical abstract

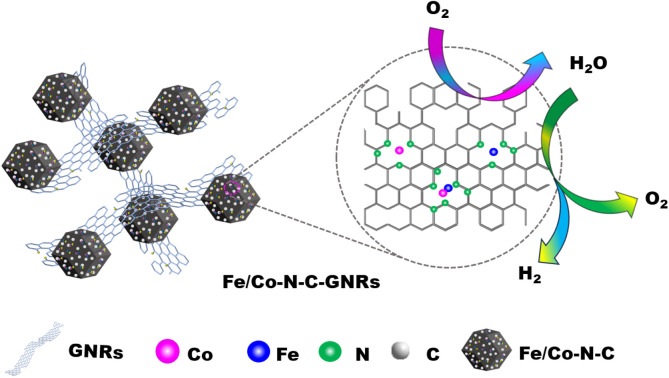

## Introduction

The development of highly efficient and environmentally friendly green energy has the potential to reduce our dependence on fossil fuels.^1–5^ Research into highly efficient functional catalytic materials for the oxygen reduction reaction (ORR) plays a significant role in the promotion of fuel cell applications.^6–12^ Recently, noble-metal-free materials, such as carbon-based porous materials codoped with transition metals (M, including iron, cobalt, nickel, and manganese) and nitrogen, are considered the most promising electrocatalysts by virtue of their low cost, high catalytic activity, and excellent stability.^13–18^ Generally, the high electrochemical activity of the catalysts is dependent on their three-dimensional (3D) porous structure facilitating rapid mass transport, multilevel interfaces, the fast diffusion of gases (such as oxygen and hydrogen), electrolytes, and abundant exposed active sites.^19,20^ Establishing a correlation between the abundant active sites and 3D porous structure of the catalyst is conducive to accelerating ORR.

Zeolitic imidazolate framework (ZIF)-67 is an excellent precursor for obtaining high-catalytic-performance M–N–C catalysts owing to its controllable 3D porous structure, mild synthetic conditions, large surface area, abundant active sites, and high Co content.^21–25^ Fe–N–C sites play a vital role in reducing the reduction of H_2_O_2_, which is a key intermediate during the O_2_ reduction process and a source of deactivation from fuel cells.^26^ However, the majority of ZIF-derived catalysts show poor electrical conductivity and narrow micropores owing to the structural collapse during high-temperature pyrolysis. Additionally, only the metal–N_*x*_ sites at the three-phase interface of carbon materials participate in the ORR as the real catalytic active sites. Structural designing, pore regulation, and prevention of the agglomeration of M–N–C catalysts remain significant challenges. Therefore, obtaining metal–N_*x*_ sites with a high-exposure morphology combined with mesopores by forming channels is necessary to increase the utilization rate of active sites and achieve good electrical conductivity.

Graphene nanoribbons (GNRs) have emerged as excellent catalyst carriers owing to their large aspect ratios, numerous edge defects, strong interactions between the metal and carrier, and special catalytic properties resulting from radial conduction.^27,28^ To increase the ORR catalytic activity of carbon materials with numerous edge defects, Tour *et al.* reported B and N-codoped GNRs exhibited the highest onset potential (1.09 V) and half-wave potential (0.96 V) for the ORR among those reported for similar catalysts. This afforded numerous active sites and accelerated the transport of oxygen and reduction products owing to a high content of B and N and multilevel porous structures of GNRs.^29^ Moreover, the transferred electrons distribute the charge density locally on the GNRs, while the positive charge is preferentially distributed on the carbon atoms near the GNR-edges or defects.^30,31^ Based on the molecular design anchoring highly graphitized carbon sheets, Lee *et al.* developed Fe–N/S-carbon nanotube-GNR catalysts containing rich defect sites and sulfur dopants, exhibiting remarkable stability during ORR.^32^ Mukerjee *et al.* reported Fe–N–C single-atom catalysts showed a high catalytic activity owing to Fe–N sites and edge sites replacing the basal plane sites on the carbon materials.^33^ However, the carbon defect sites of M–N–C single-atom catalysts could be easily oxidized, severely hindering their stability, because of the production of radicals, such as *OH or *OOH, during ORR. Therefore, the development of a preparation strategy that can expose several carbon defect sites and effectively decrease the generation of their O-containing functional groups in M–N/C single-atom catalysts is urgently required.

Herein, the presence of graphene oxide nanoribbons (GONRs) and Fe atoms on catalysts hinders agglomeration and the collapse of pores of ZIF-67 during the high-temperature pyrolysis process, respectively. Fe/Co–N–C-GNRs were calcinated from Fe/ZIF-67 particles *via in situ* growth on the surface of GONRs. Co–N–C and Fe atoms anchored on the GNRs could enhance the catalytic activity by regulating the electronic structure of the carbon surface to the d-orbital from metal sites. Fe/Co–N–C-GNRs exhibit a high electron conductivity, high electrocatalytic activity, and high durability. This is attributed to the combined coupling effects among the Fe–N_*x*_, Co–N_*x*_, FeCo–N_*x*_, and edge active sites of GNRs in Fe/Co–N–C-GNR materials. This study presents high-performance functional electrocatalysts and further provides the synthetic method for promising catalysts in fuel cell, chlor-alkali industry and lithium–oxygen battery applications.

## Results and discussion

**Scheme 1:**
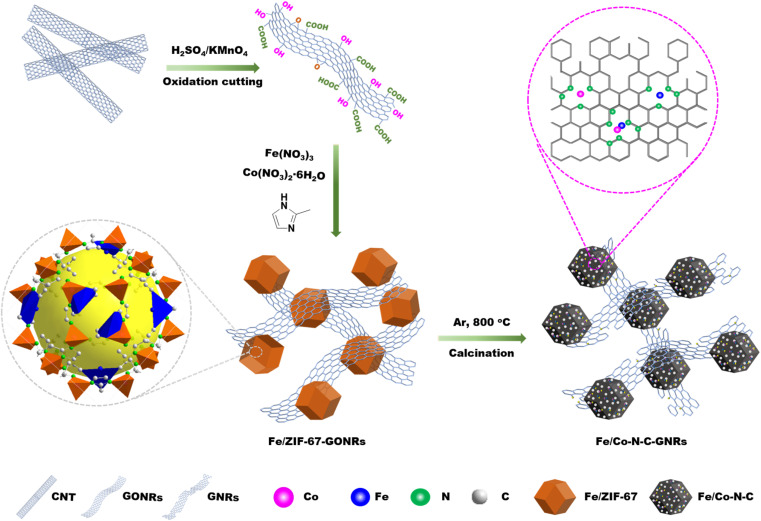
Schematic for the synthesis of Fe/Co–N–C-GNRs hybrids.

Scheme 1 shows the entire preparation process of Fe/Co–N–C-GNRs. GONRs were obtained through a previously reported method.^34^ The Co^2+^ and Fe^3+^ ions were co-added into the GONR/methanol solution and were uniformly absorbed on the surface of the GONRs. The mixture was then rapidly poured into a 2-methylimidazole/methanol solution. Subsequently, Fe/ZIF-67 was grown *in situ* on the surface of GONRs. Finally, the Fe/Co–N–C-GNRs were obtained at a high temperature (800 °C) under the Ar atmosphere.

**Fig. 1:**
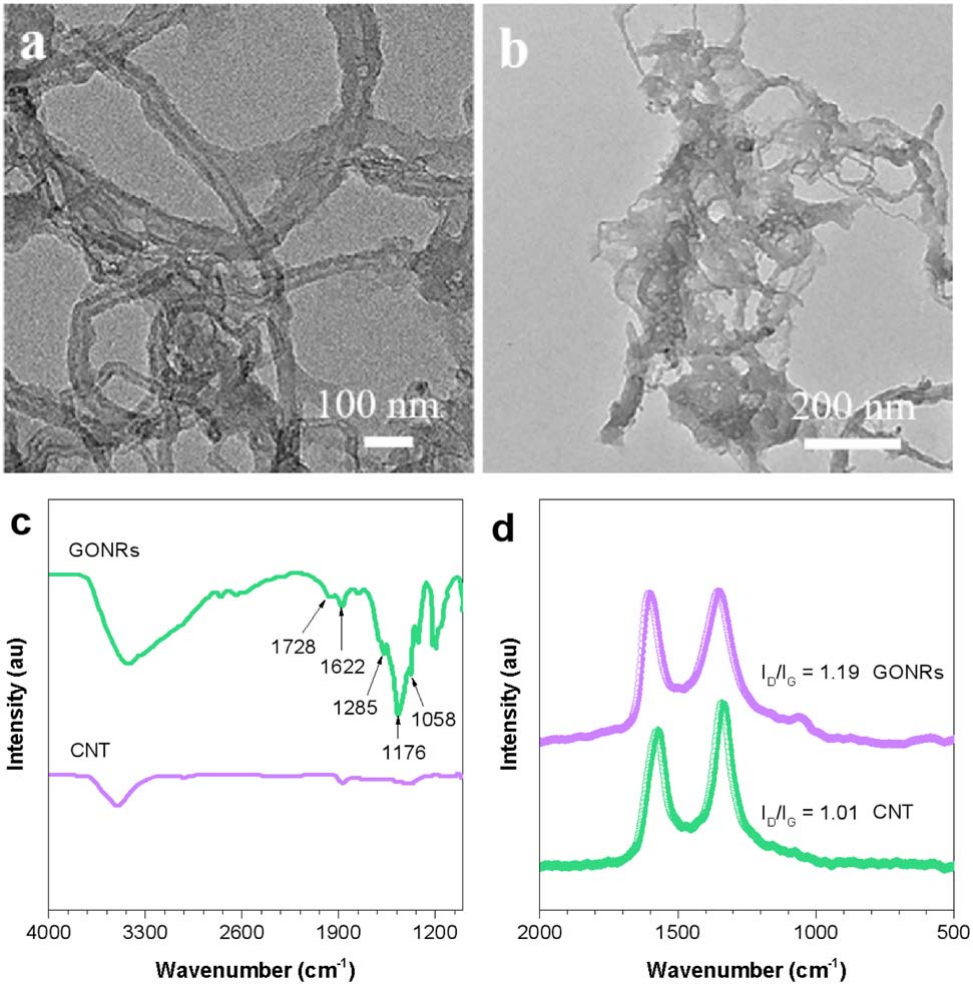
(a and b) SEM images, (c) IR and (d) Raman spectra of CNTs and GONRs.

To investigate the preparation of GONRs, transmission electron microscopy (TEM) images, infrared (IR) spectra, and Raman spectra of CNTs and GONRs were analyzed. Fig. 1a shows that the surface of CNTs is smooth, and the CNT diameter is ∼40 nm, whereas the diameter of oxidized CNTs extends to ∼80 nm after the oxidation cutting of the CNTs (Fig. 1b). Fig. 1c further shows that the IR characteristic peaks of OH and COOH groups of oxidized CNTs emerge at 1285 and 1728 cm^−1^. Emerging peaks at 1058 and 1176 cm^−1^ were also observed, which are associated with C–O–C stretching vibrations. These results indicated that the GONRs were successfully prepared from CNTs through oxidation by concentrated sulfuric acid and potassium permanganate. The as-prepared GONRs comprise rich edges and O-containing functional groups that could anchor metal ions as nucleation sites. Characteristic defect (D) and graphitic (G) peaks were observed in the Raman spectra (Fig. 1d) at 1348 and 1599 cm^−1^, respectively. The intensity ratio of the aforementioned two peaks (*I*_D_/*I*_G_) is generally used to assess the degree of carbon defect.^35–37^ The *I*_D_/*I*_G_ of the GONRs (1.19) is higher than that of the CNTs (1.01), indicating that more defects sites are present in the GONRs than those in CNTs. The defect sites in the carbon structure regulate the electronic properties and provide numerous active sites to promote the formation of nucleation sites and increase the ORR electrocatalytic activities.

After the addition of different contents, namely, 5, 10, 15, and 20 wt%, of GONRs, ZIF-67 was grown *in situ* on the surface of GONRs, to obtain ZIF-67-GONRs-5%, ZIF-67-GONRs-10%, ZIF-67-GONRs-15%, and ZIF-67-GONRs-20%, respectively, which were calcined to acquire Co–N–C-GNRs-5%, Co–N–C-GNRs-10%, and Co–N–C-GNRs-15%, and Co–N–C-GNRs-20%, respectively. To investigate the micromorphology of a series of Co–N–C-GNRs-*x* composites, those hybrids were characterised by means of scanning electron microscopy (SEM). The SEM images of ZIF-67-GONRs-5%, ZIF-67-GONRs-10%, ZIF-67-GONRs-15%, and ZIF-67-GONRs-20% (Fig. S1†) indicate that ZIF-67 is evenly dispersed depending on the content of GONRs. Fig. S1† shows particles with a regular polyhedral shape and size of ∼500 nm, indicating its successful preparation. Co–N–C shows a rough surface and collapsing carbon skeletons, indicating structural instability during the cleavage process.

**Fig. 2:**
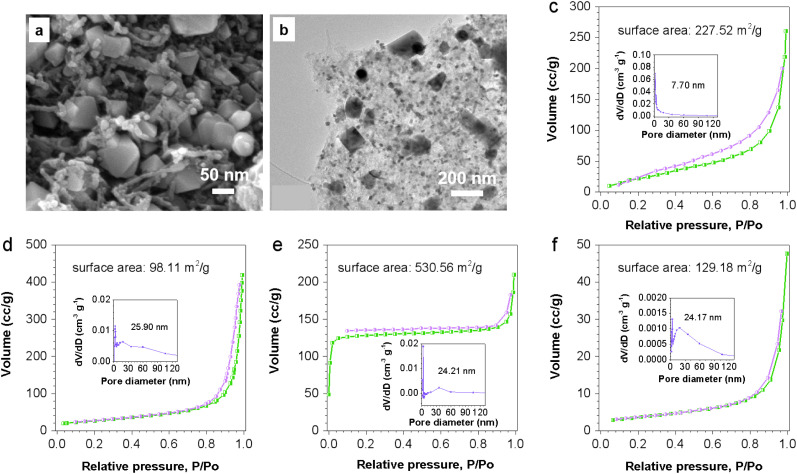
(a and b) SEM and TEM images of Fe/Co–N–C-GNRs-20% hybrids; (c–f) BET images of ZIF-67-GONRs-20%, Co–N–C-GNRs-20%, Fe/ZIF-67-GONRs-20% and Fe/Co–N–C-GNRs-20% hybrids.

The addition of GONRs prevents the agglomeration of Co–N–C in Co–N–C-GNRs-20%, and the resulting material has a more stable structure morphology than Co–N–C-GNRs-5%, Co–N–C-GNRs-10%, and Co–N–C-GNRs-15%. Therefore, the introduction of GONRs effectively prevents the aggregation of ZIF-67. To achieve more stable materials with large specific surface areas, the Fe element was added to ZIF-67-GNRs-20%. The SEM, TEM and HRTEM of Fe/ZIF-67-GONRs-20% shows a regular polyhedral morphology (Fig. 2a, b and S2†), whereas Fe/Co–N–C-GNRs-20% comprises wrinkled surfaces, less-collapsed carbon skeleton, and high dispersion of metal elements (Fe and Co), indicated that the existence of metal nitrides. The N_2_ adsorption and desorption isotherms of GONRs, Co–N–C, Co–N–C-GNRs-20%, and Fe/Co–N–C-GNRs-20% are analyzed to determine the surface area and porosity. The specific surface areas of ZIF-67 (573.1 m^2^ g^−1^) is higher than that of GONRs (153.13 m^2^ g^−1^), ZIF-67-GONRs-20% (227.52 m^2^ g^−1^), and Fe/ZIF-67-GONRs-20% (530.56 m^2^ g^−1^) (Fig. 2c, e, 4a and S3†). However, after calcination, compared with the specific surface area of Co–N–C (113.20 m^2^ g^−1^) and Co–N–C-GNRs-20% (98.11 m^2^ g^−1^) (Fig. 2d and S4b†), Fe/Co–N–C-GNRs-20% (129.18 m^2^ g^−1^) was larger (Fig. 2f), indicated that the carbon skeleton structure maintains a certain level of stability with the coaddition of GONRs and Fe elements. Therefore, Fe/Co–N–C-GNRs-20% provides numerous edge and metal–N active sites and is conducive to the fast diffusion of oxygen to effectively enhance the ORR catalytic activity.

**Fig. 3:**
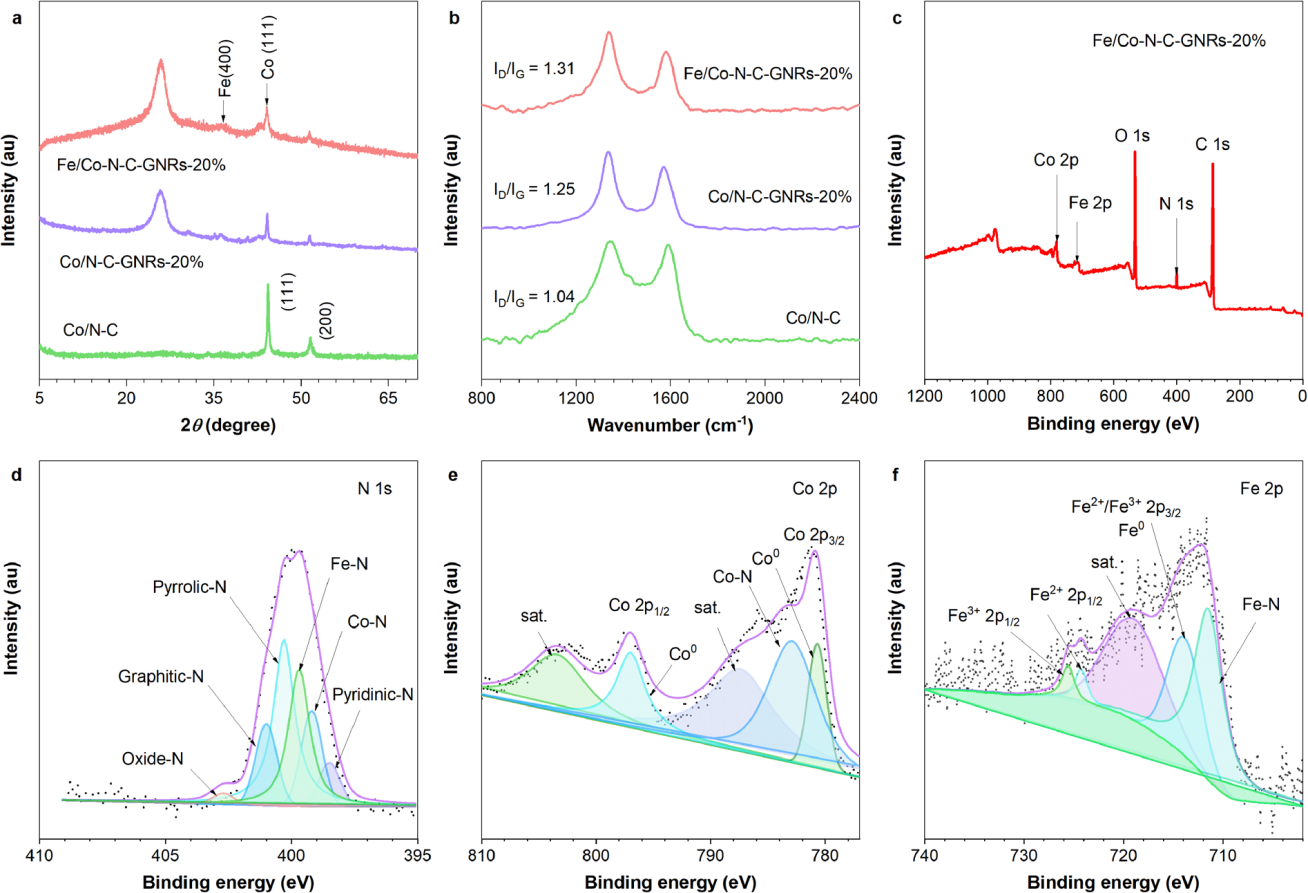
(a) XRD and (b) Raman spectra of Co–N–C, Co–N–C-GNRs-20%, and Fe/Co–N–C-GNRs-20%; (c) XPS survey scan and XPS spectra of (d) N 1s, (e) Co 2p, (f) Fe 2p for Fe/Co–N–C-GNRs-20%.

The X-ray diffraction (XRD) patterns of ZIF-67, ZIF-67-GONRs-20%, Fe/ZIF-67-GONRs-20%, Co–N–C, Co–N–C-GNRs-20%, and Fe/Co–N–C-GNRs-20% were also obtained. The peaks in the patterns of ZIF-67-GONRs-20% and Fe/ZIF-67-GONRs-20% are matched with those in the ZIF-67 patterns (Fig. S5†), indicating that ZIF-67 is successfully bound to GONRs. After high-temperature pyrolysis, two peaks were observed in the patterns of Co–N–C, Co–N–C-GNRs-20%, and Fe/Co–N–C-GNRs-20% (Fig. 3a) at 44.41° and 51.44°, which were attributed to the Co (111) and Co (200) crystal planes (PDF#150806). The peak at 25.90° belongs to the GNRs, and the peaks at 35.45° is attributed to the Fe (400) crystal planes (PDF#510740) of Fe/Co–N–C-GNRs-20%, suggesting the successful introduction of Co, Fe, and GNRs in these composites. Furthermore, the Raman spectra of Co–N–C, Co–N–C-GNRs-20%, and Fe/Co–N–C-GNRs-20% were characterized to calculate their edge site degrees. The *I*_D_/*I*_G_ ratio of Fe/Co–N–C-GNRs-20% hybrids is 1.31 and is higher than those of Co–N–C (1.04) and Co–N–C-GNRs-20% (1.25) (Fig. 3b), indicating that more edge sites exist in the Fe/Co–N–C-GNRs-20% than those in the other samples. These results confirm the presence of numerous catalytic active sites, such as Fe–N_*x*_, Co–N_*x*_, FeCo–N_*x*_, and GNR edge sites, for increasing the ORR catalytic activity and mass transfer capacity of oxygen.

To further identify chemical elements with different compositions and chemical states, the X-ray photoelectron (XPS) spectra of Co–N–C-GNRs-20% and Fe/Co–N–C-GNRs-20% composites were analyzed (Fig. 3c–f and S6†). Fig. 3c–f and Table 1 present the different elements and contents of Co–N–C-GNRs-20%, and Fe/Co–N–C-GNRs-20%. The high-resolution N 1s spectrum of Fe/Co–N–C-GNRs-20% (Fig. 3d) comprises peaks corresponding to six entities: pyridine-N (398.5 eV), graphitic-N (401 eV), pyrrolic-N (400.3 eV), Co–N (399.7 eV), Fe–N (399.2 eV), and oxizied-N (403 eV). The pyridinic-N and graphitic-N sites can improve the ORR catalytic activity because the metal–N_*x*_ active sites may be derived from the pyridine-N bonds of the carbon materials.^38^ According to the XPS high-resolution spectrum of N 1s, the oxizied-N content of Fe/Co–N–C-GNRs-20% (1.64%) is significantly lower than that of Co–N–C-GNRs-20% (28.42%) (Fig. 3e, f and Table 2), indicating a higher utilization of the N element, as oxizied-N is not effective for ORR.

**Table 1:** The different elemental content of Co–N–C-GNRs-20% and Fe/Co–N–C-GNRs-20% by XPS

Samples	C 1s (%)	N 1s (%)	O 1s (%)	Co 2p (%)	Fe 2p (%)
Co–N–C-GNRs-20%	91.43	4.43	2.52	1.62	—
Fe/Co–N–C-GNRs-20%	78.40	2.14	14.53	1.77	3.16

**Table 2:** The different type-N content of Co–N–C-GNRs-20% and Fe/Co–N–C-GNRs-20% by XPS

Samples	Pyridine-N (%)	Graphitic-N (%)	Pyrrolic-N (%)	Fe–N_*x*_ (%)	Co–N_*x*_ (%)	Oxizied-N (%)
Co–N–C-GNRs-20%	11.37	25.58	20.42	—	14.21	28.42
Fe/Co–N–C-GNRs-20%	6.54	12.26	36.79	14.31	28.46	1.64

As seen in Table 1, both Co–N–C-GNRs-20% and Fe/Co–N–C-GNRs-20% have a high content of N elements, encompassing Co–N_*x*_ and Fe–N_*x*_ species, which can provide numerous catalytic active sites and high mass transfer efficiency for enhancing the ORR performance. The Co–N_*x*_ and Fe–N_*x*_ sites play a key role in enhancing the effective ORR catalytic activity.^39^ The Co 2p spectrum of Fe/Co–N–C-GNRs-20% manifests the existence of Co–N (782.9 eV) and Co^0^ (780.7 eV and 797.0 eV) (Fig. 3e), respectively. The Fe 2p spectra (Fig. 3f) exhibit Fe–N (711.4 eV) and Fe^0^ (714 eV) peaks, which further confirms that numerous Fe–N_*x*_ active sites are present in Fe/Co–N–C-GNRs-20%. These results indicate that a large number of Co–N_*x*_, Fe–N_*x*_, pyridinic-N, and graphitic-N active sites coexist in Fe/Co–N–C-GNRs-20%, which are beneficial to enhance the ORR catalytic activity. The presence of a significant amount of oxygen content in the catalyst materials, as indicated by XPS data, could have several potential influences on the ORR catalysis. These oxygens could be some oxygen-containing functional groups residual on the GONRs and air, such as hydroxyl (–OH) or carbonyl (–C=O) groups, could serve as active sites for the ORR. These groups can facilitate the adsorption and activation of oxygen molecules.

To study the effect of the GNRs content on the ORR performance, the cyclic voltammetry (CV) measurements of Co–N–C-GNRs-5%, Co–N–C-GNRs-10%, Co–N–C-GNRs-15%, and Co–N–C-GNRs-20% were performed with a scan rate of 100 mV s^−1^ in an O_2_-saturated alkaline solution. Fig. S7 and S8† shows the cathodic reduction peak (0.84 V) of Co–N–C-GNRs-20%. CV is more positive than that of Co–N–C-GNRs-5% (0.62 V), Co–N–C-GNRs-10% (0.82 V), and Co–N–C-GNRs-15% (0.81 V). Moreover, the *J*_L_ values of Co–N–C-GNRs-20% were superior to those of other composites (Fig. S9–S12†), indicating that Co–N–C-GNRs-20% exhibit better ORR catalytic activity depending on the GNR content, compared to the other samples. This is attributed to the rich active sites at the edges of GNRs,^40^ GNRs with abundant edge sites could efficiently increase the chemisorption of oxygen to increase the catalytic activity of Co–N–C-GNRs-20%.

**Fig. 4:**
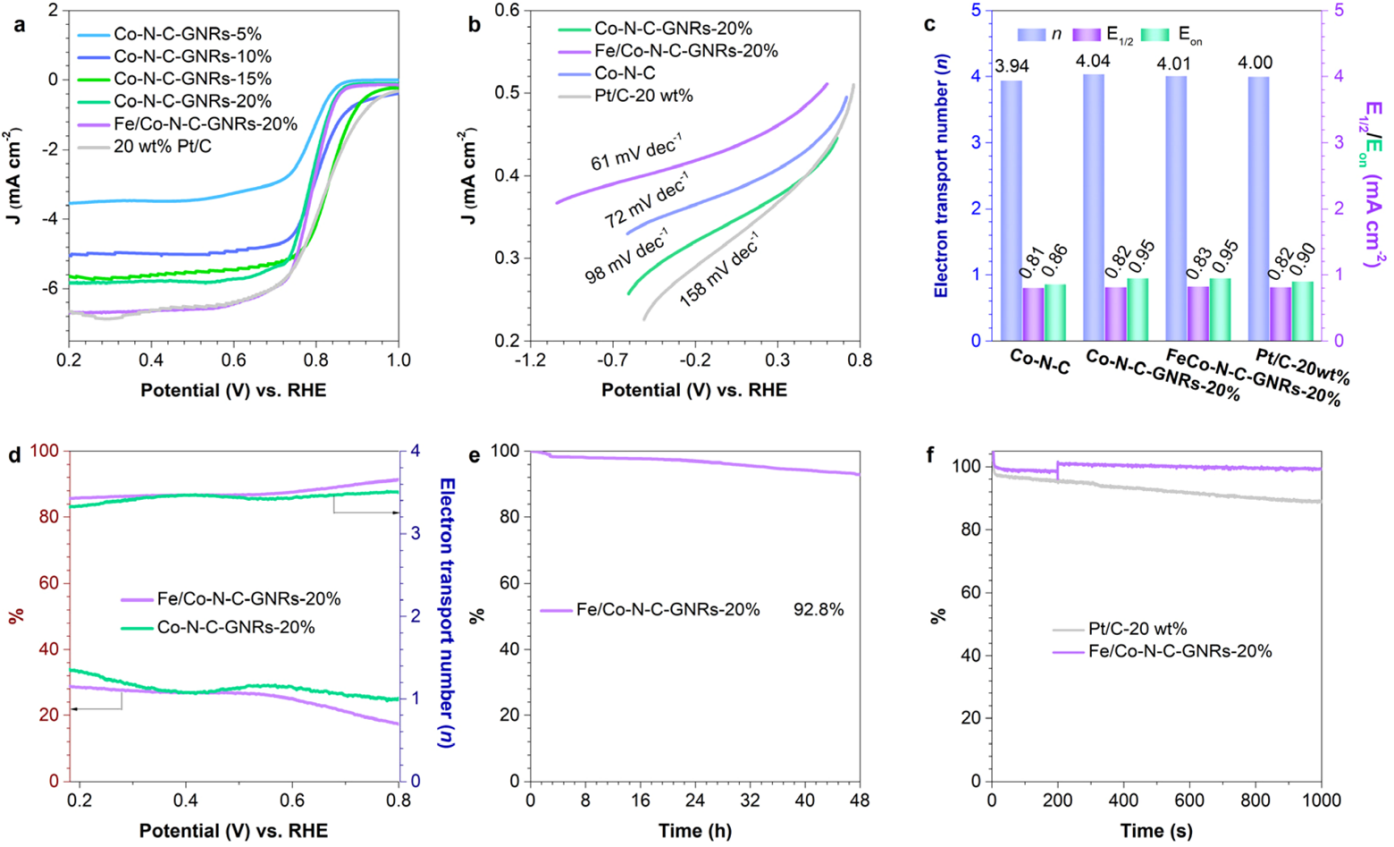
(a) LSV curves of Co–N–C-GNRs-5%, Co–N–C-GNRs-10%, Co–N–C-GNRs-15%, Co–N–C-GNRs-20%, Fe/Co–N–C-GNRs-20%, and Pt/C-20 wt%, (b) Tafel plots and (c) on set potential (*E*_on_), half-wave potential (*E*_1/2_), n of Co–N–C, Co–N–C-GNRs-20%, Fe/Co–N–C-GNRs-20%, and Pt/C-20 wt%, (d) H_2_O_2_ yield and *n* curves of Co–N–C-GNRs-20% and Fe/Co–N–C-GNRs-20% on a RRDE at 1600 rpm during ORR. Under a potential of 0.83 V, (e) *i*–*t* chronoamperometric response for 48 h of Fe/Co–N–C-GNRs-20% and (f) methanol tolerance with 3 wt% methanol addition at around 200 s of Co–N–C-GNRs-20%, Fe/Co–N–C-GNRs-20%, and Pt/C-20 wt%.

To comprehensively clarify the factors affecting the ORR performance of a series of catalysts, the electrochemical properties of Fe/Co–N–C-GNRs-20%, and 20 wt% Pt/C were also characterized. In the LSV profile, Fe/Co–N–C-GNRs-20% shows a highly positive onset potential (*E*_on_) of 0.95 V and a half-wave potential (*E*_1/2_) of 0.83 V during the linear sweep voltammetry (LSV) measurements (Fig. 4a, S13 and 14†), and these values are superior to the results obtained for Co–N–C (0.86 and 0.81 V), Co–N–C-GNRs-20% (0.95 and 0.82 V), 20 wt% Pt/C (0.90 and 0.82 V) (Fig. 4a, c and Table 3), and several other previously reported M/N_*x*_–C-carbon materials (Table S1†).^41–47^ Moreover, Fe/Co–N–C-GNRs-20% exhibited a high current density of 6.70 mA cm^−2^ (*J*_L_, Fig. 4c and Table 3), which was higher than those of Co–N–C-GNRs-20% (5.69 mA cm^−2^) and 20 wt% Pt/C (6.63 mA cm^−2^). The Tafel slope of Fe/Co–N–C-GNRs-20% was lower at 63 mV dec^−1^ (Fig. 4b), compared with the Tafel slopes obtained for Co–N–C-GNRs-20% (67 mV dec^−1^) and 20 wt% Pt/C (170 mV dec^−1^). Furthermore, the electrochemical impedance spectrum (EIS) of Fe/Co–N–C-GNRs-20% is conducted, indicating that the material containing only one semi-circle is free of ionomers and R1 is the diffusion resistance.^48,49^ This reveals low diffusion resistance of Fe/Co–N–C-GNRs-20% enhances material diffusion, leading to better catalytic performance. (Fig. S15 and Table S2†). These results therefore suggest that Fe/Co–N–C-GNRs-20% is a highly efficient ORR catalyst in KOH aqueous solution, further revealing the structure–activity correlation among the edge, M–N_*x*_ sites, and catalytic activity.

**Table 3:** ORR activity comparison for Co–N–C, Co–N–C-GNRs-20%, Fe/Co–N–C-GNRs-20% and Pt/C-20 wt%

Samples	*E*_on_ a (V *vs.* RHE)	*E*_1/2_ b (V *vs.* RHE)	*J*_L_ c	*n* d
Co–N–C	0.86	0.81	5.96	3.93
Co–N–C-GNRs-20%	0.95	0.82	5.69	4.04
Fe/Co–N–C-GNRs-20%	0.95	0.83	6.70	4.15
Pt/C-20 wt%	0.90	0.82	6.63	4.00

a *E*_on_ = on set potential.

b *E*_1/2_ = half-wave potential.

c *J*_L_ = limiting current density (mA cm^−2^).

d *n* = electron transport number.

The selectivity of the electrocatalysts in the ORR process was further studied. The yield of H_2_O_2_ in the ORR process was recorded *via* the rotating ring-disk electrode (RRDE) test, to examine the ORR pathway (Fig. 4d and S17†). Fe/Co–N–C-GNRs-20% showed a H_2_O_2_ yield of <20%. Additionally, the *n* value of Fe/Co–N–C-GNRs-20% was ∼3.86 within the potential range of 0.2–0.8 V, indicating an unconventional selectivity of oxygen reduction to OH– through the 4-electron pathway, which is well matched with the calculation results of Fe/Co–N–C-GNRs-20% (∼4.0) at all potentials obtained using the Koutecký–Levich (K–L) plots (Fig. 4c and S15†) and is closed to that of Pt/C-20 wt%. Meanwhile, Co–N–C-GNRs-20% and Co–N–C exhibit low selectivity in the four-electron pathway.

The electrochemical stability of Fe/Co–N–C-GNRs-20% was further studied *via* the chronoamperometry tests at a potential of 0.83 V. Fe/Co–N–C-GNRs-20% exhibits a current retention of 92.8% after 48 h (Fig. 4e), which is better than that of Co–N–C-GNRs-20% (78.8%) and 20 wt% Pt/C (86.9%) after 10 000 s (Fig. S18†). In addition, Fe/Co–N–C-GNRs-20% shows an excellent methanol resistance along with negligible current decay after adding methanol into the alkaline aqueous solution (Fig. 4f). These results confirm that Fe/Co–N–C-GNRs-20% possesses high durability and an excellent methanol resistance.

The Fe/Co–N–C-GNRs-20% exhibit high ORR catalytic activity. This can be attributed to the following elements: (1) the incorporation of iron in ZIF-67 enables the preservation of porosity during calcination. A large number of Co–N_*x*_, Fe–N_*x*_, and FeCo–N_*x*_ sites are generated and exposed, thereby enhancing the ORR catalytic activity; (2) the incorporation of GNRs not only provides abundant edge sites for increasing the ORR catalytic activity but also inhibits the aggregation of ZIF-67 nanoparticles during pyrolysis. Moreover, GNRs possess superior electrical conductivity which further improves mass transfer efficiency; (3) the introduction of the nitrogen element effectively modulates the spin and charge density within the carbon structure facilitating enhanced oxygen adsorption and ORR activity. These unique features collectively endow Fe/Co–N–C-GNRs-20% with exceptional electrocatalytic activity, excellent durability and superior methanol resistance.

**Fig. 5:**
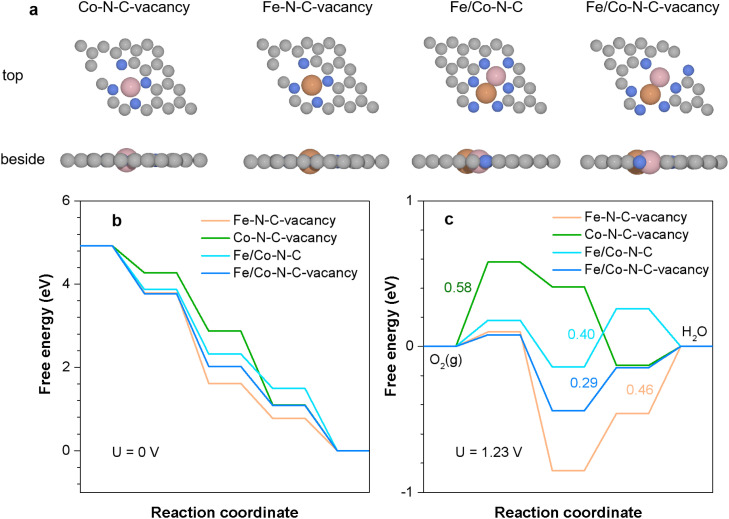
Density functional theory calculations. (a) Four types of models and (b and c) calculated free energy diagrams of the ORR on Co–N–C-vacancy, Fe–N–C-vacancy, FeCo–N–C, and FeCo–N–C-vacancy.

To further explore the origin of the electrocatalytic activity of Fe/Co–N–C-GNRs-20%, density functional theory (DFT) calculations were carried out. There are four types of models designed of Fe/Co–N–C-GNRs-20% (Fig. 5a), due to the coaddition of Fe and GNR vacancy sites. The calculated free energy diagrams for the ORR catalytic activity on Co–N–C-vacancy, Fe–N–C-vacancy, FeCo–N–C, and FeCo–N–C-vacancy are shown in Fig. 5b and c. Additionally, detailed information regarding the optimized geometry of Co–N–C-vacancy, Fe–N–C-vacancy, FeCo–N–C, and FeCo–N–C-vacancy with adsorbed intermediates 
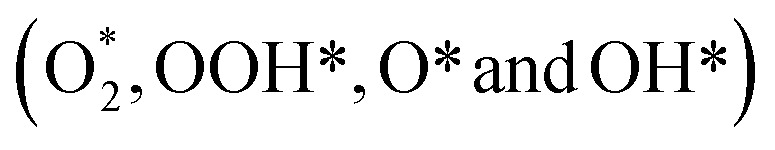
 is also provided (Tables S3 and S4†). The free energies at the 0 electrode potential of Co–N–C-vacancy, Fe–N–C-vacancy, FeCo–N–C, and FeCo–N–C-vacancy exhibit a pronounced downward trend, indicating a thermodynamically spontaneous exothermic process.^50^ When the equilibrium potential is 1.23 V, there are several endothermic steps captured in the four models.

From Fig. 5c, it can be observed that FeCo–N–C-vacancy shows the lowest endothermic energy (0.29 eV), which is lower than that of FeCo–N–C (0.40 eV). Higher endothermic energies of 0.58 and 0.46 eV were observed for Co–N–C-vacancy and Fe–N–C-vacancy, respectively. The lower theoretical limiting potential indicates that the catalytic activity of FeCo–N–C-vacancy is superior in alkaline media compared with that of FeCo–N–C (which displays more sluggish kinetics). Therefore, these results clarified that Fe/Co–N–C-GNRs-20% possess exceptional ORR electrocatalytic activity, revealing that the introduction of Fe and GNR defects can improve the adsorption/desorption behavior of adsorbed oxygen-containing intermediates by inducing a spatial redistribution of charge around the active site, thereby affecting the catalytic properties.

## Conclusions

In summary, Fe/Co–N–C-GNRs-20% was easily synthesized by loading porous N-doped carbon nanoparticles with abundant edge defects, as well as Fe–N_*x*_, and Co–N_*x*_, and FeCo–N_*x*_ sites on GONRs. The coaddition of Fe and GONRs effectively increases the number of catalytic active sites in Fe/Co–N–C-GNRs-20%. Fe/Co–N–C-GNRs-20% exhibits better ORR catalytic activity (*E*_on_ = 0.95 V_RHE_, *E*_1/2_ = 0.83 V_RHE_, and high durability (89.9%)) than Pt/C-20 wt%. These results confirmed that combined coupling effects among the Fe–N_*x*_, Co–N_*x*_, FeCo–N_*x*_, and GNR edge active sites of Fe/Co–N–C-GNRs-20% materials are main reasons for functional electrocatalytic performances. This study provides a facile preparation strategy for promising potential catalysts that can be applied in fuel cells, chlor-alkali industry lithium–oxygen batteries.

## Author contributions

This work was finished through contributions of all authors. The preparation of Fe/Co–N–C-GNRs-20% used in this work, most of tests in electrochemical properties were contributed by Xiao Hong Chen, Qiao Wu, Yupan Zhang, Junchao Xiong. With the help of Di Ma, Xiaoyu Xie, the fitting lines of XRD and XPS were successfully completed. Prof. Jun Lin, Dr Hongjie Meng, and Prof. Ying Wu directed the whole process in this system and revised manuscript. All authors have given approval to the final version of this manuscript.

## Conflicts of interest

The authors declare no conflict of interest.

## Supplementary Material

RA-015-D4RA07513G-s001

## Data Availability

The data supporting this article have been included as part of the ESI.†
