# Supplementary material for: Combined effects of transition metal nitrogen and graphene nanoribbon edge active sites on the oxygen reduction reaction catalytic performance of metal–N–carbon-based catalysts
Source: RSC Adv. 2025 Jun 20;15(26):20863–71. doi: 10.1039/d4ra07513g (PMC12179858; doi:10.1039/d4ra07513g)
Supplement: RA-015-D4RA07513G-s001 [file RA-015-D4RA07513G-s001.pdf]

Supplementary Information (SI) for RSC Advances.
This journal is © The Royal Society of Chemistry 2025

Combined effects of transition metal nitrogen and graphene nanoribbon edge active sites on the oxygen reduction reaction catalytic performance of metal-N-carbon-based catalysts

Xiaohong Chen,[a] Qiao Wu,[a,b] Yupan Zhang,[a,b] Junchao Xiong,[c] Di Ma,[a,b] Xiaoyu Xie,[a,b] Jun Lin,[c] Ying Wu,[a,*] Hongjie Meng[d,*]

[a] Institute of Energy Power Innovation, North China Electric Power University, Beijing 102206 China

[b] School of Energy, Power and Mechanical Engineering, North China Electric Power University, Beijing, 102206 China

[c] School of New Energy, North China Electric Power University, Beijing, 102206 China

[d] School of Energy Science and Technology, Longzihu New Energy Laboratory, Henan University, Zhengzhou, 450046, China,

E-mail: yingwu2000@hotmail.com; menghongjie@sjtu.edu.cn

Part 1: Experiments

1.1 Materials

Multi-carbon nanotubes were purchased from Nanjing XFNANO Materials Tech Co., Ltd. Potassium permanganate, concentrated sulfuric acid (98 %), methanol (99.9 %), potassium hydroxide (90 %), ferric nitrate nonahydrate (98 %), 2-methylimidazole, and cobalt (II) nitrate hexahydrate were purchased from Shanghai Titan Technology Co., Ltd. Potassium hydroxide was obtained from Sinopharm Chemical Reagent Co., Ltd.

1.2 Apparatus

The morphology and structure were analyzed by scanning electron microscopy (SEM) (Tescan Mira 3xh) and transmission electron microscopy (TEM) (Jeol JEM2100). The surface characteristics and composition were determined by X-ray diffraction (XRD) (Smart Lab). The elemental composition were investigated by X-ray photoelectron spectroscopy (XPS) (Thermo Scientific Escalab 250XI). The defect degree and graphitization degree were analyzed by Raman spectroscopy (Thermo DXR 2XI). Quadrasorb EVO was used to measure the specific surface area and pore size distribution at a temperature of 77.3 K, based on the N_2 adsorption-desorption isotherm. The specific surface area and pore size distribution were calculated by Brunauer Emmet-Teller (BET) method and Barrett-Joyner-Halenda (BJH) model. Cyclic voltammograms were collected in a three-electrode system (glass carbon electrode (GCE) or modified GCE as the working electrode, a Pt wire as counter electrode, and a reversible hydrogen electrode

(RHE) as the reference) at room temperature. For rotating disk electrode (RDE) measurements, a glassy carbon rotating disk electrode was used as the working electrode. Linear sweep voltammetry was performed at the GC disk electrode. In all of the electrochemical measurements, 0.1 M KOH aqueous solution saturated with nitrogen (N_2) or oxygen (O_2) was used as the electrolyte.

1.3 Synthetic procedures

1.3.1 Synthesis of graphene oxide nanoribbons (GONRs)

One gram of multiwalled carbon nanotubes (MWCNTs) were added to 130 mL of concentrated H_2SO_4 with stirring, and after 1 h, 15 mL of 30 wt% H_2O_2 was added with stirring. Thereafter, the obtained suspension was filtered and washed, and the process was repeated for three times. The black viscous solid was placed into a vacuum oven and dried at 50 ^oC for 24 h to obtain GONRs.

1.3.2 Synthesis of Zeolitic imidazolate framework (ZIF)-67/GONRs-5%, ZIF-67/GONRs-10%, ZIF-67/GONRs-15%, ZIF-67/GONRs-20%, and Fe/ZIF-67/GONRs-20%

First, the preparation of A solution: GONRs (246 mg) and were added to methanol (20 mL) dispersion with stirring for 30 min, and then cobalt nitrate hexahydrate (300 mg) was also added to the A solution with stirring for 1 h. The preparation of B solution: 2-methylimidazole (684 mg) was dissolved in methanol (21 mL) and stirred for 10 min. The B solution was added quickly to the A solution and stirred for 24 h. Final, the products were washed and dried to obtain ZIF-67/GONRs-20%.

According to the above procedures, ZIF-67 was obtained by removing GONRs.

GONRs was added with different contents of GONRs, in order to obtain ZIF-67/GONRs-5%, ZIF-67/GONRs-10% and ZIF-67/GONRs-15%. Fe/ZIF-67/GONRs-20% was synthesized by adding 50 mg of ferric nitrate nonahydrate.

1.3.3 Synthesis of Co-N-C, Co-N-C-GNRs-5%, Co-N-C-GNRs-10%, Co-N-C-GNRs-15%, Co-N-C-GNRs-20%, and Fe/Co-N-C-GNRs-20% composites

The obtained ZIF-67, ZIF-67/GONRs-5%, ZIF-67/GONRs-10%, ZIF-67/GONRs-15%, ZIF-67/GONRs-20%, and Fe/ZIF-67/GONRs-20% composites were pyrolyzed at 800 ^{o}C in inert gas for 2 h to obtain Co-N-C, Co-N-C-GNRs-5%, Co-N-C-GNRs-10%, Co-N-C-GNRs-15%, Co-N-C-GNRs-20%, and Fe/Co-N-C-GNRs-20% composites respectively.

The details steps of materials physical characterizations, electrochemical and water splitting measurements, and density functional theory method are given in Supporting Information.

1.4 Cyclic voltammograms (CV), ring-disk electrode (RDE) and linear sweep voltammetry (LSV) and electrochemical impedance spectroscopy (EIS) measurements

A conventional cell with a three-electrode configuration was applied throughout this work. CV measurement was conducted at 25 ^{o}C using an Autolab PGSTAT302 (Metrohm) electrochemical and Shanghai Chenhua Electrochemical Workstation test system using RHE as the reference electrode, a Pt wire as the counter electrode and

the sample modified GCE as the working electrode. 4 μL of 1 mg mL^{-1} ink (1 mg sample, 10 μL Nafion 117 solution, 0.2 mL ethanol and 0.8 mL Millipore water) was loaded on a glassy carbon RDE (φ = 3 mm). 0.1 M KOH aqueous solution was used as the electrolyte, which was saturated with O_2 by bubbling it prior to the start of each experiment. A flow of O_2 was maintained over the electrolyte during the recording of CVs in order to ensure continuous O_2 saturation. In control experiments, CV measurements were performed under N_2 atmosphere. 0.1 M KOH aqueous solution saturated with nitrogen or oxygen was used as the electrolyte in all electrochemical measurements.

Cyclic Voltammetry (CV) and Linear Sweep Voltammetry (LSV) tests were performed at 10 mV s^{-1} scanning rate. The voltage range of CV test was -1 to 0.25 V and the rotating speeds was controlled from 400 rpm to 2000 rpm. The applied potential vs. Ag/AgCl (3 M KCl) was converted to reversible hydrogen electrode (RHE) potential using the following equation:

$$E_{RHE} = E_{Ag/AgCl} + 0.059pH + E^{\circ}_{Ag/AgCl}$$

where $E_{Ag/AgCl}$ is the experimentally measured potential using Ag/AgCl as the reference electrode and $E_{Ag/AgCl}$ is 0.210 V.

The electron transfer number can be calculated by Koutecky- Levich (K-L) equations:

$$\frac{1}{J} = \frac{1}{J_L} + \frac{1}{J_K} = \frac{1}{B\omega^{1/2}} + \frac{1}{J_K}$$

$$B = 0.62nFC_0(D_0)^{2/3}v^{-1/6} \quad J_k = nFkC_0$$

where J is the measured current density, J_K and J_L are the kinetic- and diffusion

limiting current densities, ω is the angular velocity of the rotating electrode, F is the Faraday constant (F = 96485 C mol^{-1}), C_0 is the concentration of O_2 in 0.1 M KOH (1.2×10^{-3} M), D_0 is the diffusion coefficient of O_2 in 0.1 M KOH (1.9×10-5 cm^2 s^{-1}), ν is the kinematic viscosity of the electrolyte (0.01 cm^2 s^{-1}), and k is the electron transfer rate constant.

Tafel slopes were calculated according to the Tafel equation:

$$\eta = b\,log\,(j) + a$$

where η, j and b are the over potential, measured current density, and Tafel slope, respectively. Moreover, rotating ring-disk electrode (RRDE) of the catalyst was performed at 1600 rpm in 0.1 M KOH medium, the values of n and H_2O_2 yields could be determined according to the following equations:

$$H_2O_2\% = 100 \times \frac{2I_r/n}{I_d + I_r/N}$$

$$n = \frac{4I_d}{I_d + I_r/N}$$

where I_d and I_r are disk current and ring current, respectively, N is the H_2O_2 collection efficiency at the ring.

The methods for stability and methanol tolerance measurements

(1) Stability tests were performed at 10 mV s^{-1} scanning rate in 0.1 M KOH under the potential at 0.62 V and the rotating speeds was controlled at 1600 rpm for 172800 s (48 h).

(2) The methanol tolerance tests were performed at 10 mV s^{-1} scanning rate in 0.1 M KOH under the potential at 0.62 V and the rotating speeds was controlled at 1600 rpm. Methanol solution (3 wt%) was added into the 0.1 KOH solution at 200 s.

The methods for EIS measurements

The EIS measurements of Fe/Co-N-C-GNRs-20% were conducted in nitrogen-saturated 0.1 M KOH, at $E_{1/2}$ = 0.83 V. The impedance data were collected using an Autolab PGSTAT302 (Metrohm) and a Shanghai Chenhua Electrochemical Workstation, configured in a three-electrode thin-film rotating disk electrode setup. Impedance spectra were acquired by sweeping the frequency from 10 kHz to 50 mHz with 10 data points per decade, utilizing an AC perturbation amplitude of 5 mV rms. All measurements were performed under single-sine waveform excitation. The obtained impedance spectra were analyzed and fitted using the complex non-linear least squares method with the "ZSimpWin" software package from Solartron.

2 Density functional theory method

The DFT calculations were conducted using the Vienna ab initio Simulation Package (VASP) with the implementation of projector augmented wave (PAW). The exchange and correlation potential was calculated using the generalized gradient approximation (GGA) because incorporating gradient corrections to the local spin density approximation is essential for accurately describing the magnetic properties of iron.

Part 2: Figures

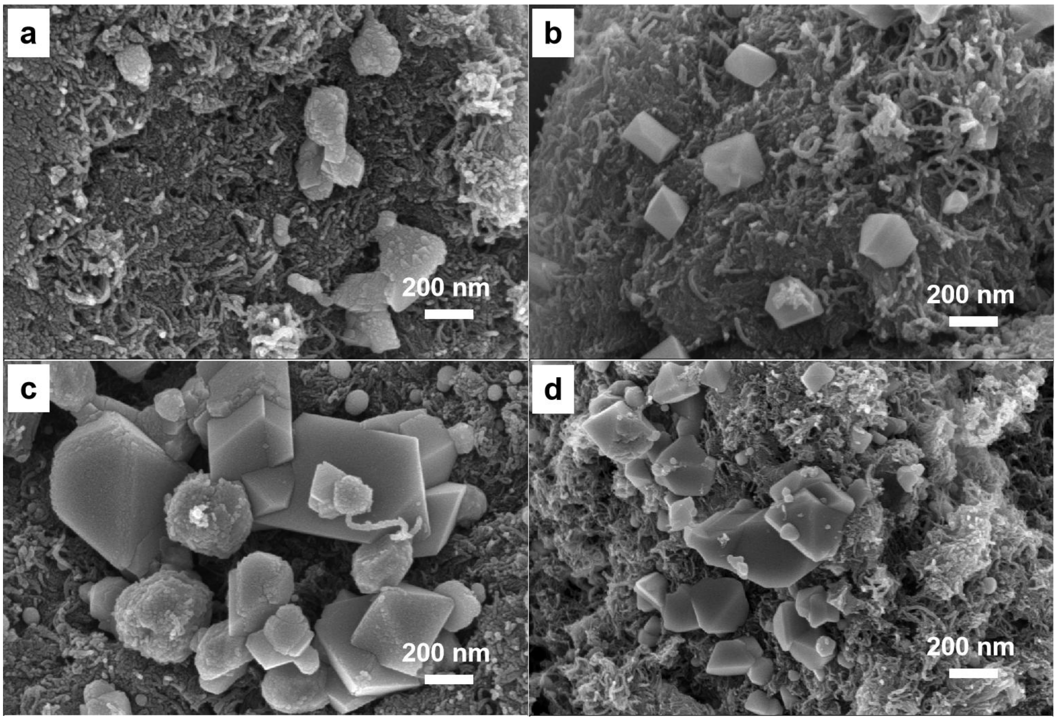

Fig. S1. SEM images of (a) ZIF-67-GONRs-5%, (b) ZIF-67-GONRs-10%, (c) ZIF-67-GONRs-15% and (d) ZIF-67-GONRs-20%.

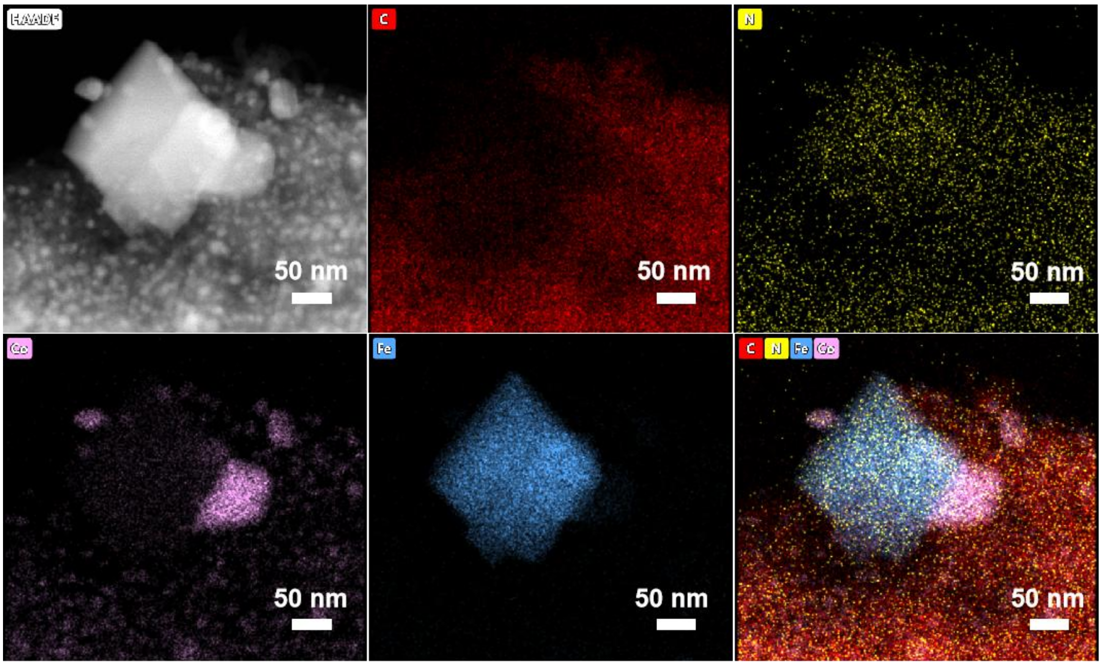

Fig. S2. The TEM mapping of Fe/Co-N-C-GNRs-20%.

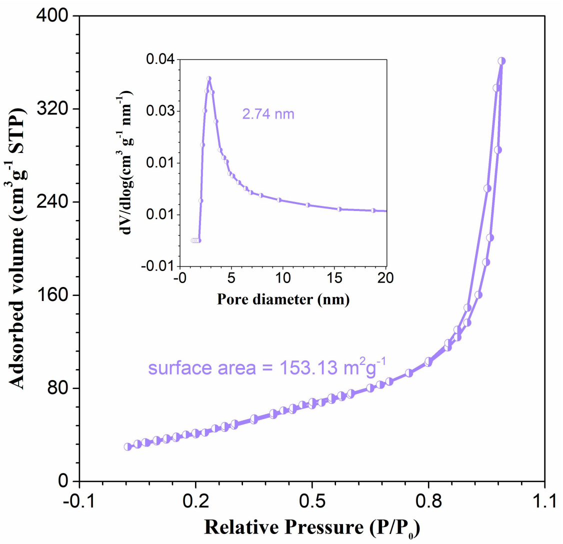

Fig. S3. BET spectra and pore size of the GONRs.

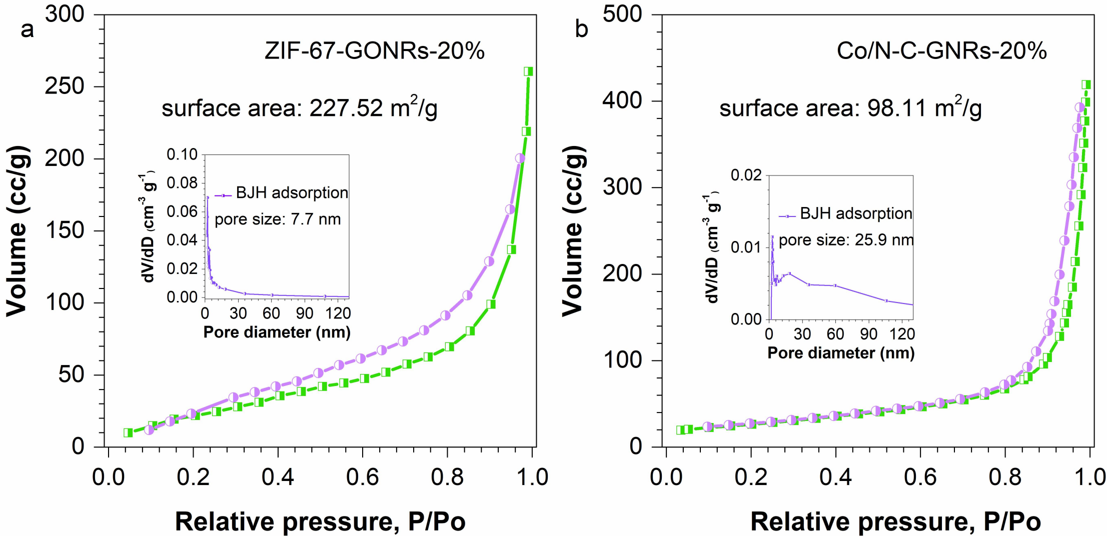

Fig. S4. N_2 adsorption/desorption isotherms and pore size distribution of (a) ZIF-67 and (b) Co-N-C.

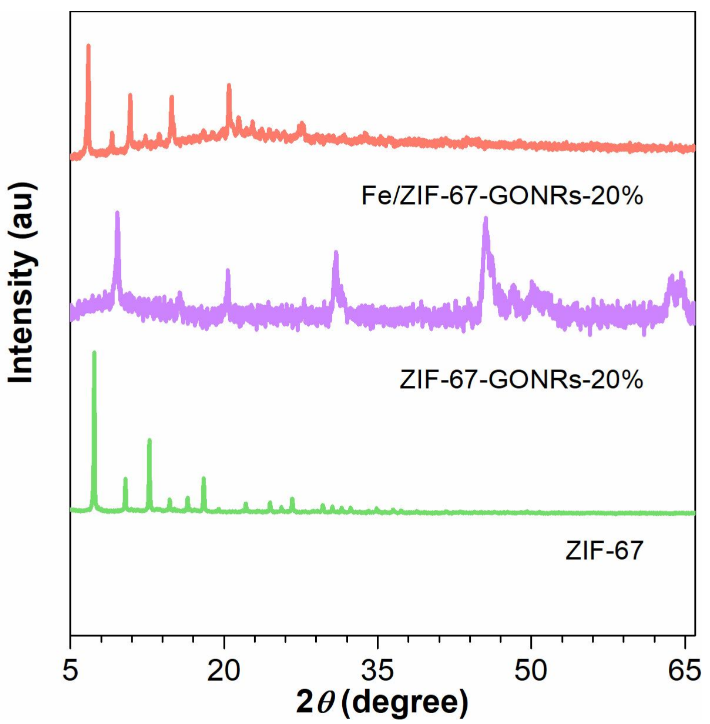

Fig. S5. XRD spectra of ZIF-67, ZIF-67-GONRs-20% and Fe/ZIF-67-GONRs-20%.

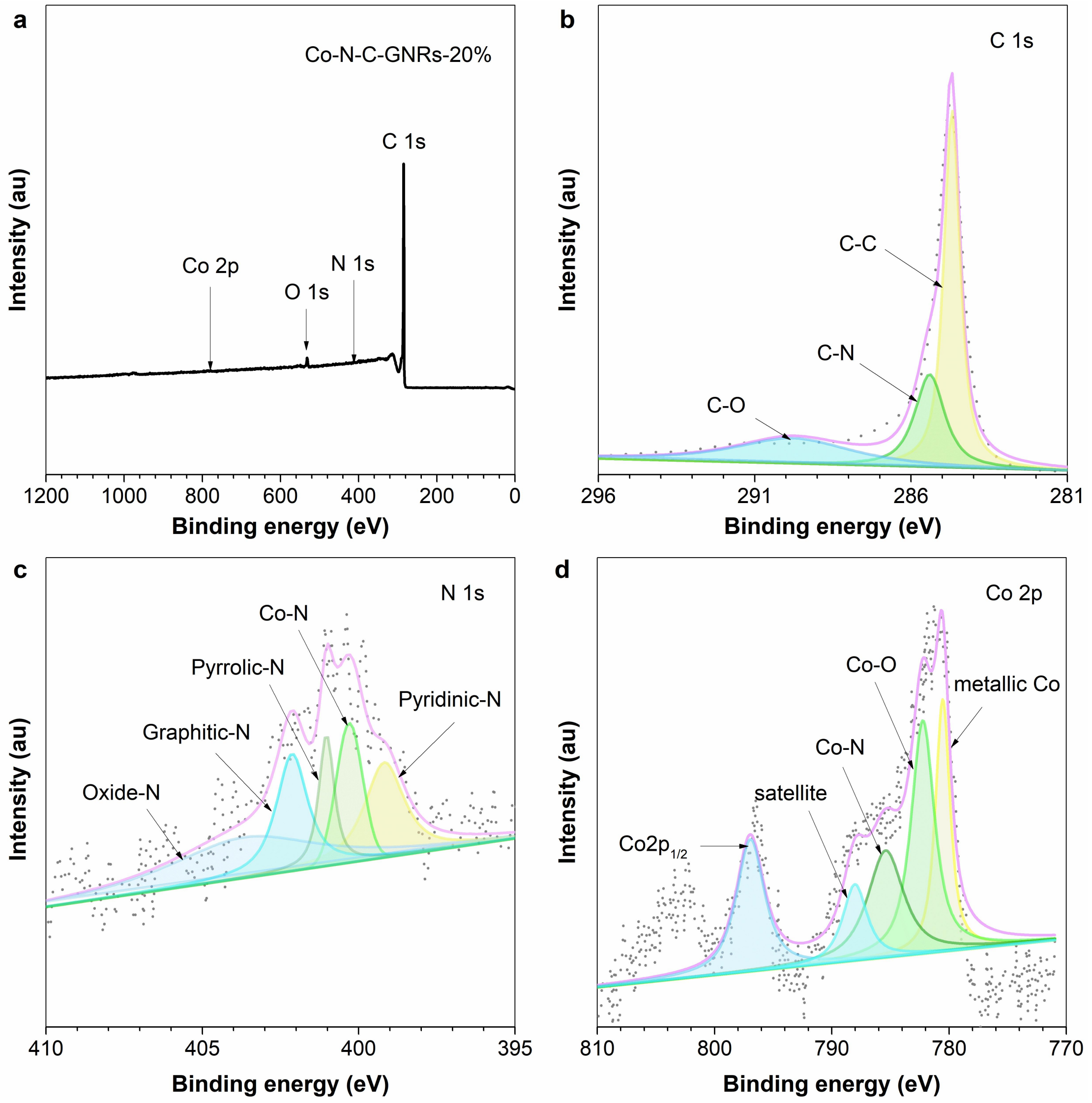

Fig. S6. The XPS spectra of (a) survey, (b) C 1s, (c) N 1s and (d) Co 2p for Co-N-C-GNRs-20%.

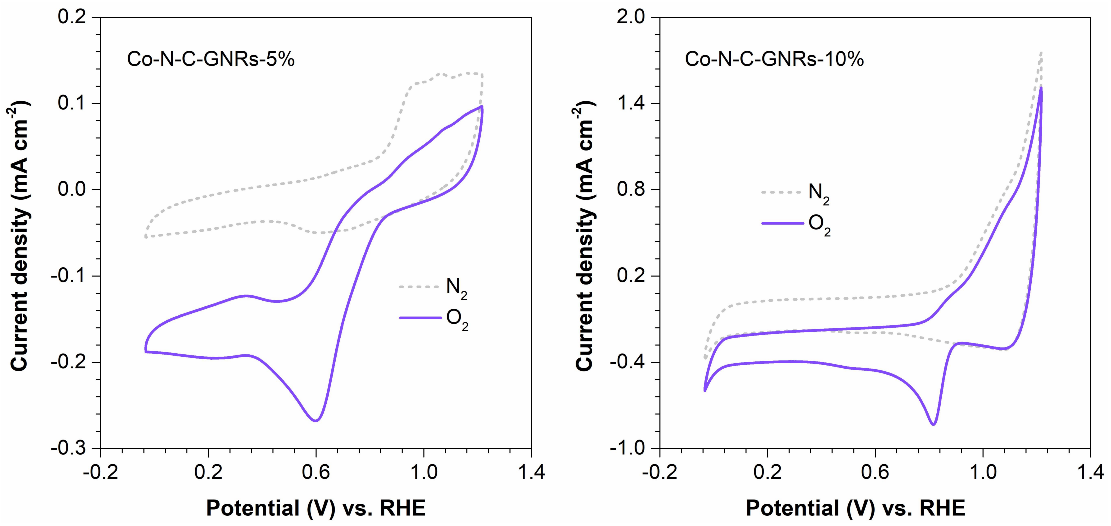

Fig. S7. CV curves of Co-N-C-GNRs-5% and Co-N-C-GNRs-10%.

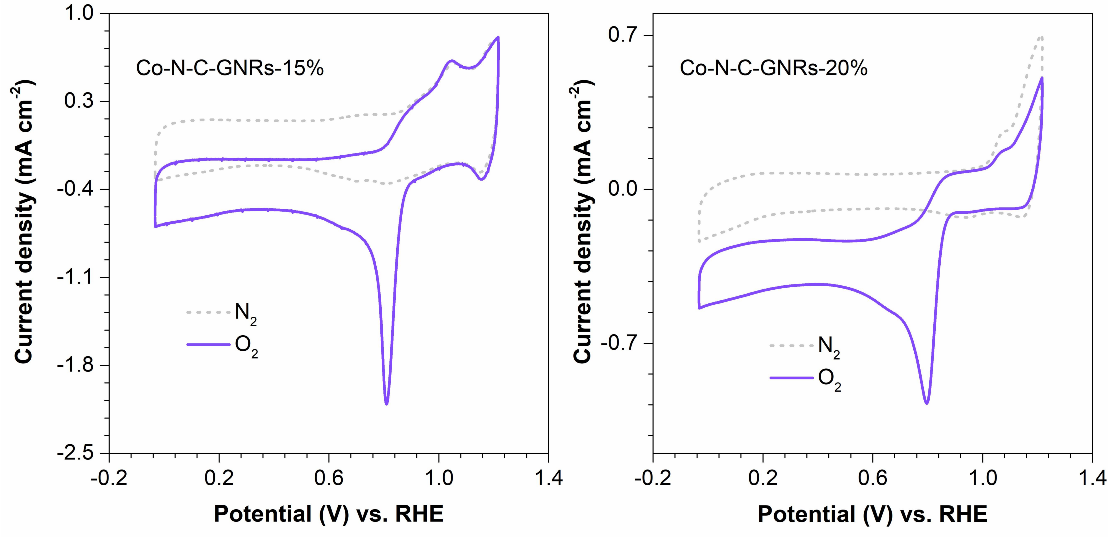

Fig. S8. CV curves of Co-N-C-GNRs-15% and Co-N-C-GNRs-20%.

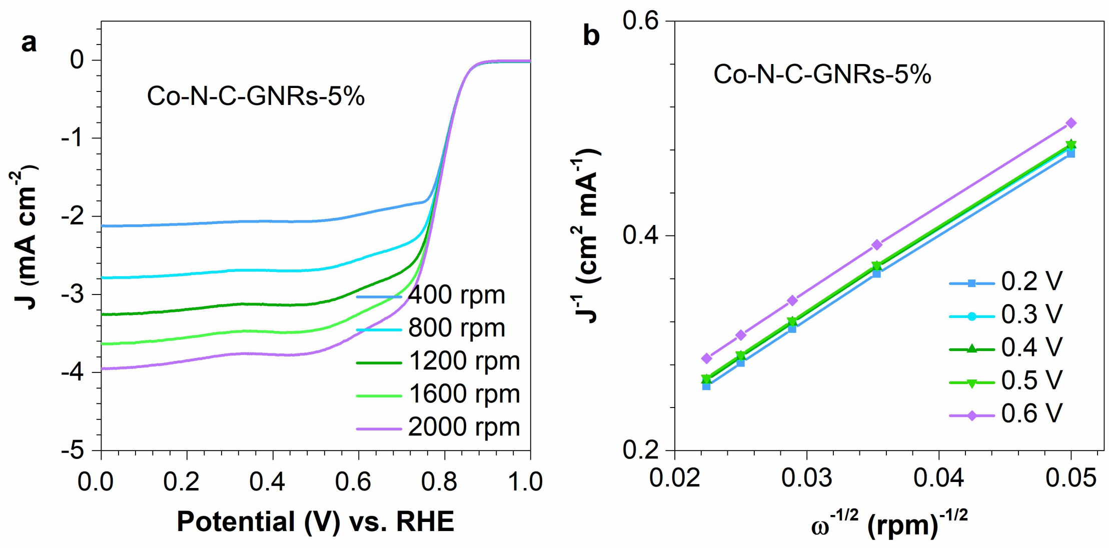

Fig. S9. (a) LSV curves at different rotating speeds in O_2-saturated 0.1 M KOH solution and (b) corresponding K−L plots of Co-N-C-GNRs-5% under different potentials.

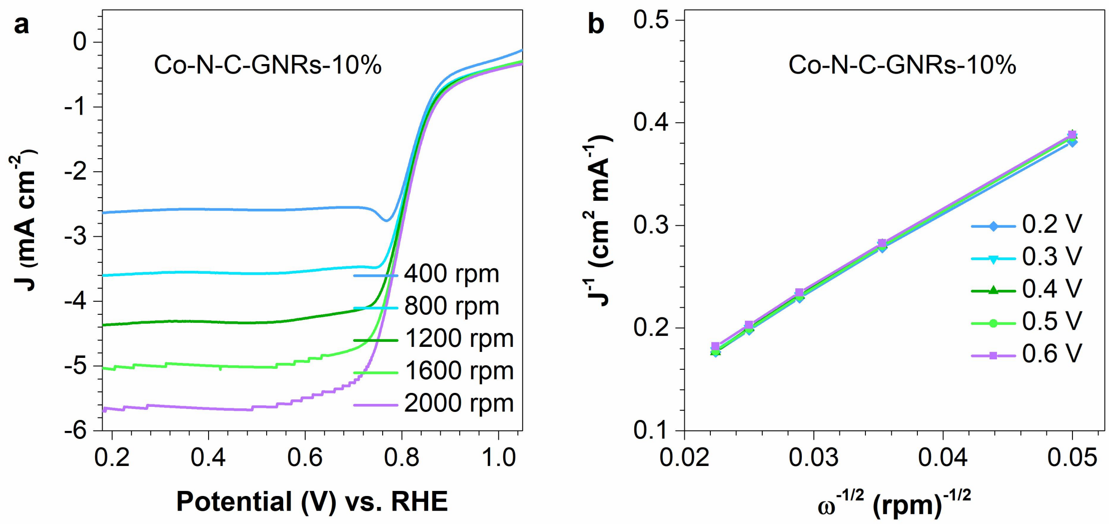

Fig. S10. (a) LSV curves at different rotating speeds in O_2-saturated 0.1 M KOH solution and (b) corresponding K−L plots of Co-N-C-GNRs-10% under different potentials.

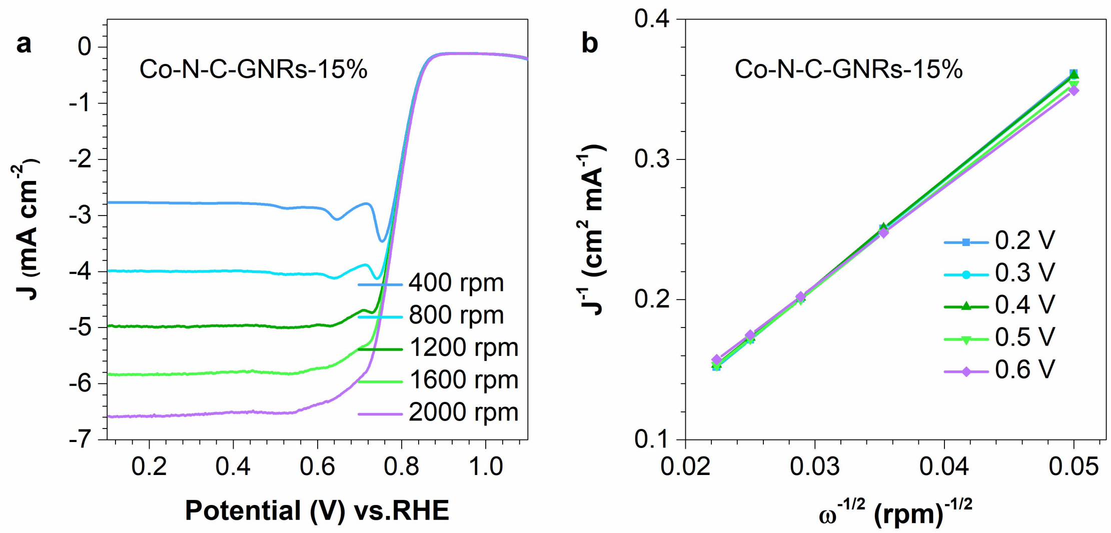

Fig. S11. (a) LSV curves at different rotating speeds in O_2-saturated 0.1 M KOH solution and (b) corresponding K−L plots of Co-N-C-GNRs-15% under different potentials.

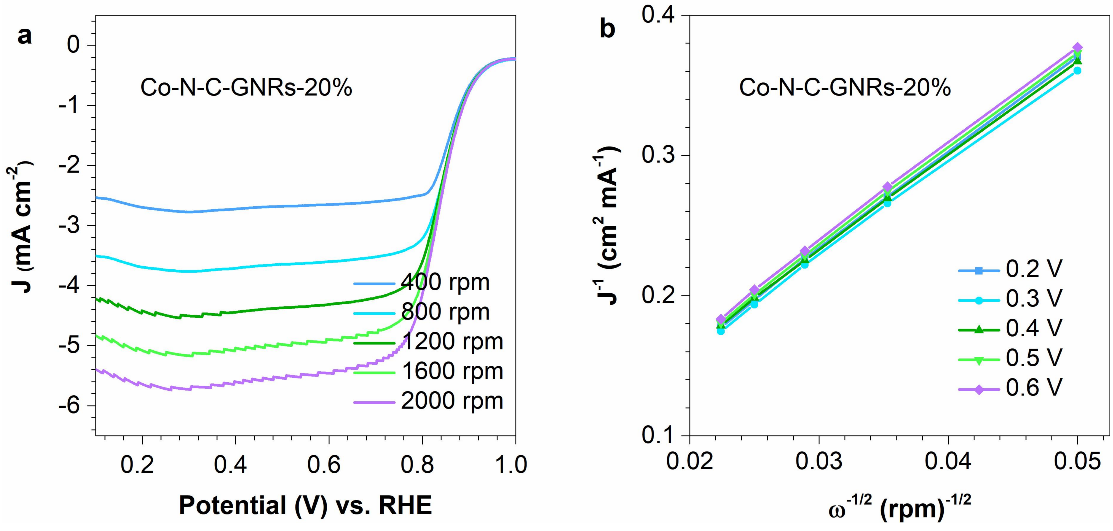

Fig. S12. (a) LSV curves at different rotating speeds in O_2-saturated 0.1 M KOH solution and (b) corresponding K−L plots of Co-N-C-GNRs-20% under different potentials.

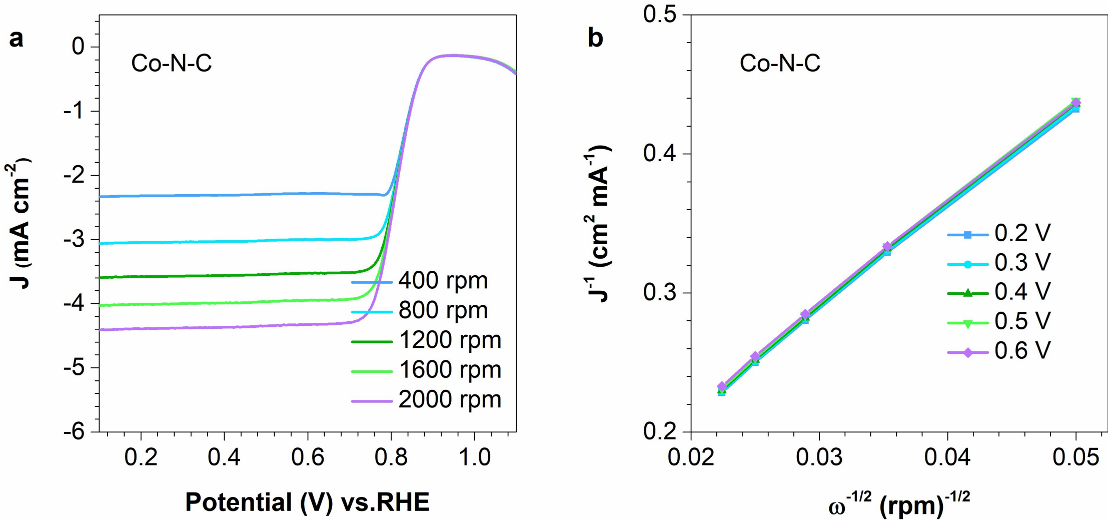

Fig. S13. (a) LSV curves at different rotating speeds in O_2-saturated 0.1 M KOH solution and (b) corresponding K-L plots of Co-N-C under different potentials.

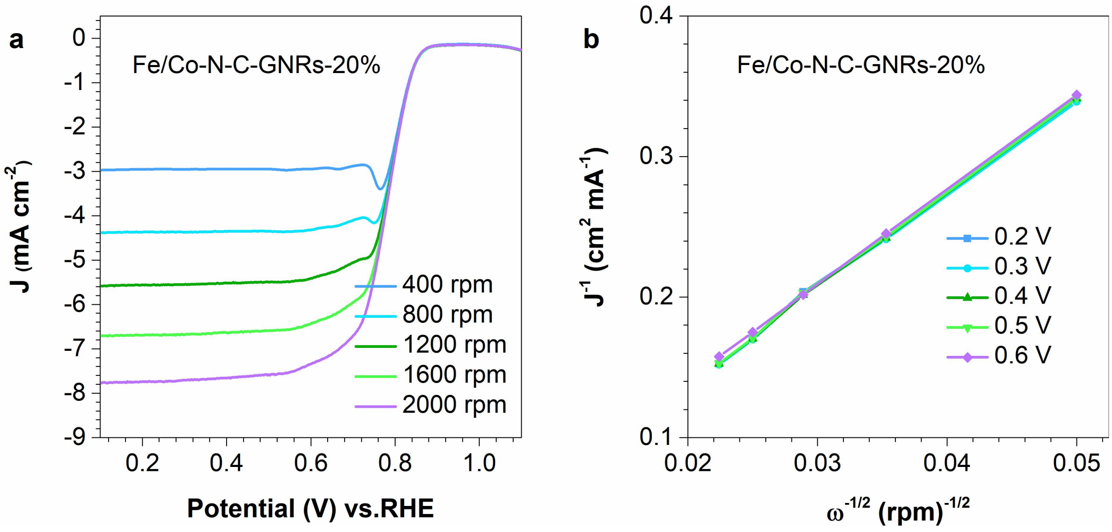

Fig. S14. (a) LSV curves at different rotating speeds rotating speeds in O_2-saturated 0.1 M KOH solution and (b) corresponding K−L plots of Fe/Co-N-C-GNRs-20% under different potentials.

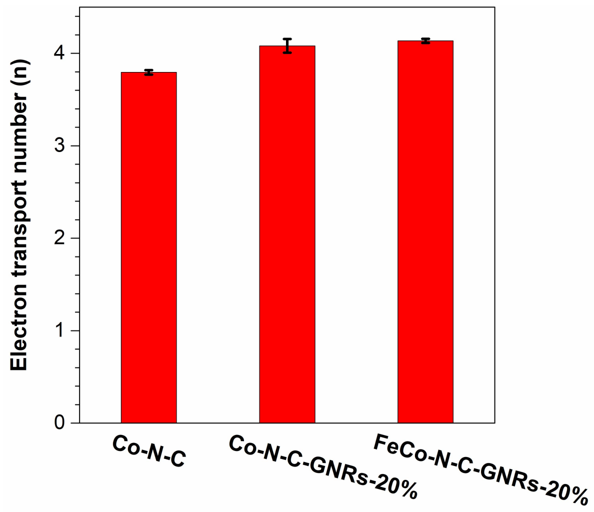

Fig. S15. Electron transport number (n) of Co-N-C, Co-N-C-GNRs-20% and Fe/Co-N-C-GNRs-20%. (The source of the error bars: the the number of electron transfers for Co-N-C, Co-N-C-GNRs-20% and Fe/Co-N-C-GNRs-20% was calculated using K-L plots at 0.2 V, 0.3 V, 0.4 V, 0.5 V, and 0.6 V. The error bar plot was constructed based on the different numbers of electron transfers obtained at these five voltages.)

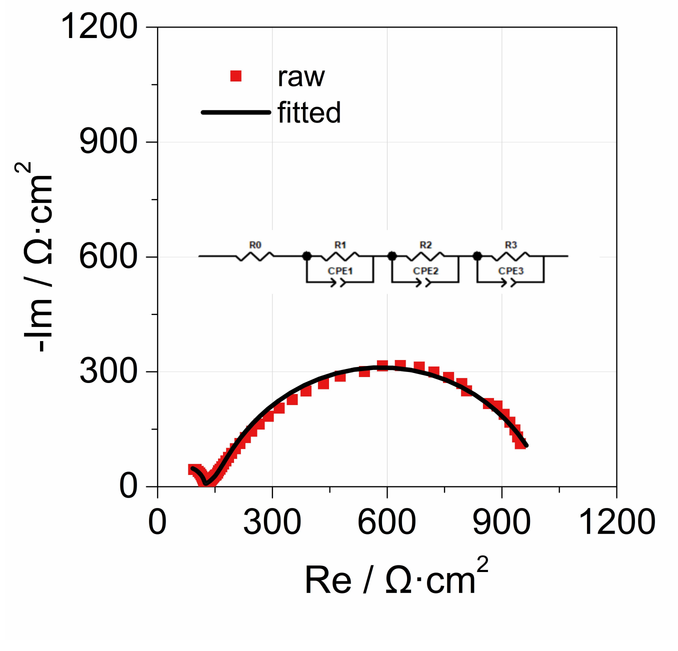

Fig. S16. The EIS spectra of Fe/Co-N-C-GNRs-20%.

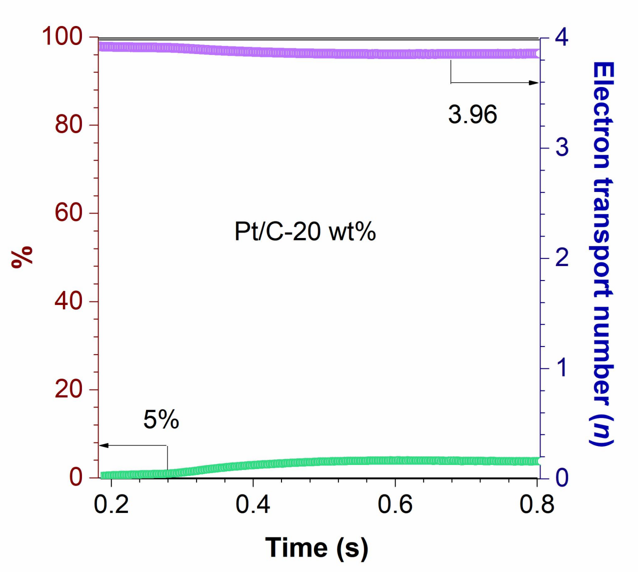

Fig. S17. H_2O_2 yield and n curves of Pt/C-20 wt% on a RRDE at 1600 rpm during ORR.

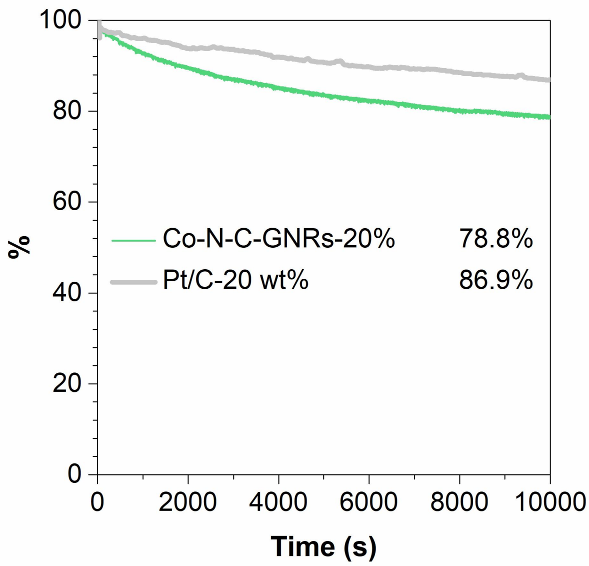

Fig. S18. Under a potential of 0.83 V, i-t chronoamperometric response for 10000 s of Co-N-C-GNRs-20%, and Pt/C-20 wt %.

Because the current density remains stable during the potential range of 0.2 V-0.6 V, these five voltage points are selected for calculating the electron transfer number.

Table S1 Comparison of the ORR catalytic performances of Fe/Co-N-C-GNRs with those of other ZIF-derived heteroatom-doped carbon catalysts reported in literature.

Samples	E_{onset} (V vs RHE)	$E_{1/2}$ (V vs RHE)	J_L (mA cm^{-2})	n	Ref
**Fe/Co-N-C-GNRs**	**0.95**	**0.83**	**6.7**	**4.15**	**This work**
Fe/Co-N-C-graphene	0.91	0.85	6.7	4.02	41
Co@O-NPC-700	--	0.8	5.3	4.00	42
Fe/N-PCNs	--	0.86	--	3.95	43
Co_3O_4/HNCP-40	--	0.84	5.8	3.90	44
FeCo/Co_2P@NPCF	0.85	0.79	4.8	3.85	45
Co@N-C-1	1.02	0.93	4.5	3.95	46
Co-N-PCN	0.90	0.82	--	4.00	47

Table S1 The different EIS values of Fe/Co-N-C-GNRs-20%.

R0 (Ω)	R1 (Ω)	R2 (Ω)	R3 (Ω)
1.8715E+1	1.0284E+2	3.5426E+1	8.5948E+2

Table S3 DFT parameters of calculated Ezpe and TS for H_2 and H_2O at 293 K.

parameters	H_2	H_2O
ZPE	0.273	0.541
TS	0.401	0.579

Table S4 DFT parameters of calculated Ezpe and TS for Fe-N-C-vavancy, Co-N-C-vavancy, FeCo-N-C and FeCo-N-C-vavancy at 293K.

Systems	parameters	*OH	*O	*OOH
Fe-N-C-vavancy	ZPE	0.33	0.05	0.39
	TS	0.11	0.08	0.14
Co-N-C-vavancy	ZPE	0.33	0.04	0.32
	TS	0.08	0.06	0.21
FeCo-N-C	ZPE	0.31	0.04	0.36
	TS	0.07	0.07	0.17
FeCo-N-C-vavancy	ZPE	0.28	0.05	0.43
	TS	0.06	0.06	0.18

Reference

[1] J. K. Nørskov, J. Rossmeisl, A. Logadottir, L. Lindqvist*,* Origin of the overpotential for oxygen reduction at a fuel-cell cathode. J Phys Chem B **2004**, *108*, 17886–17892.
